# Update on the Preventive Antibiotics in Stroke Study (PASS): a randomised controlled phase 3 clinical trial

**DOI:** 10.1186/1745-6215-15-133

**Published:** 2014-04-21

**Authors:** Willeke F Westendorp, Jan-Dirk Vermeij, Nan van Geloven, Diederik WJ Dippel, Marcel GW Dijkgraaf, Tom van der Poll, Jan M Prins, Lodewijk Spanjaard, Frederique H Vermeij, Paul J Nederkoorn, Diederik van de Beek

**Affiliations:** 1 Department of Neurology, Academic Medical Centre, Amsterdam, The Netherlands; 2 Clinical Research Unit (CRU), Academic Medical Centre, Amsterdam, The Netherlands; 3 Department of Neurology, Erasmus MC University Medical Centre, Rotterdam, The Netherlands; 4 Centre of Infection and Immunity (CINIMA), Academic Medical Centre, Amsterdam, The Netherlands; 5 Department of Infectious Diseases, Academic Medical Centre, Amsterdam, The Netherlands; 6 Department of Microbiology, Academic Medical Centre, Amsterdam, The Netherlands; 7 Department of Neurology, Sint Franciscus Gasthuis, Rotterdam, The Netherlands; 8 Department of Neurology, H2.216, Academic Medical Centre, PO box 22660, Amsterdam 1100 DD, The Netherlands

**Keywords:** Stroke, Infection, Antibiotics

## Abstract

**Background:**

Stroke is a leading cause of death worldwide. Infections after stroke occur in 30% of stroke patients and are strongly associated with unfavourable outcome. Preventive antibiotic therapy lowers infection rate in patients after stroke, however, the effect of preventive antibiotic treatment on functional outcome after stroke has not yet been investigated.The Preventive Antibiotics in Stroke Study (PASS) is an ongoing, multicentre, prospective, randomised, open-label, blinded end point trial of preventive antibiotic therapy in acute stroke. Patients are randomly assigned to either ceftriaxone at a dose of 2 g, given every 24 hours intravenously for four-days, in addition to stroke-unit care, or standard stroke-unit care without preventive antibiotic therapy. Aim of the study is to assess whether preventive antibiotic treatment improves functional outcome at three months by preventing infections.

**Results:**

To date, 2,470 patients have been included in PASS. Median stroke severity of the first 2,133 patients (second interim analysis) is 5 (IQR 3 to 9) on the National Institutes of Health Stroke Scale (NIHSS). Due to the PROBE design, no outcome data are available yet. In the initial trial protocol we proposed a dichotomisation of the mRS as primary analysis of outcome and ordinal regression analysis as secondary analysis of primary outcome, requiring a sample size of 3,200 patients. However, ordinal analysis of outcome data is becoming increasingly more common in acute stroke trials, as it increases statistical power. For PASS, funding is insufficient for inclusion of 3,200 patients with the overall inclusion rate of 15 patients per week. Therefore we change the analysis of our primary outcome from dichotomisation to ordinal regression analysis on the mRS. Power analysis showed that with similar assumptions 2,550 patients are needed using ordinal regression analysis. We expect to complete follow-up in June 2014. A full statistical analysis plan will be submitted for publication before treatment allocation will be unblinded.

**Conclusion:**

The data from PASS will establish whether preventive antibiotic therapy in acute stroke improves functional outcome by preventing infection. In this update, we changed our primary outcome analysis from dichotomisation to ordinal regression analysis.

**Trial registration:**

Current controlled trials; ISRCTN66140176. Date of registration: 6 April 2010.

## Update

### Preventive Antibiotics in Stroke Study (PASS)

Stroke is a leading cause of death worldwide. Infections after stroke occur in 30% of stroke patients and are strongly associated with unfavourable outcome [1,2]. Preventive antibiotic therapy lowers infection rate in patients after stroke; however, the effect of preventive antibiotic treatment on functional outcome after stroke has not yet been investigated [3,4].

The aim of PASS is to investigate whether preventive use of the antibiotic ceftriaxone improves functional outcome in patients with stroke. PASS is an ongoing, multicentre Prospective, Randomised, Open-label, Blinded End point trial (PROBE) of standard care with preventive ceftriaxone treatment which is compared with standard care without preventive ceftriaxone. Adult patients with stroke (both ischaemic and haemorrhagic) and a score ≥ 1 on the National Institutes of Health Stroke Scale will be included. Patients are randomly assigned to either ceftriaxone at a dose of 2 g, given every 24 hours intravenously for four-days, in addition to stroke-unit care, or standard stroke-unit care without preventive antibiotic therapy. All items from the World Health Organization Trial Registration Data Set are shown in Table 1. For description of the entire study protocol, including study procedures and data collection, assessment of infections and outcomes, allocation and blinding procedures, we refer to the initial trial protocol publication [5]. Changes to the protocol since the first version are shown in Table 2. Medical-ethical approval of the protocol and amendments was obtained by the medical ethical committee of the AMC. All participating centres are shown in Table 3.

**Table 1:** All items from the World Health Organization Trial Registration Data Set (SPIRIT checklist, item 2b)

**Data category**	**Information**
Primary registry and trial identifying number	Current controlled trials; http://www.controlled-trials.com; ISRCTN66140176
Date of registration in primary registry	6 April 2010
Secondary identifying numbers	-
Source(s) of monetary or material support	1. Netherlands Organisation for Health Research and Development (ZonMw) (Netherlands) (ref: 171002302) 2. Netherlands Heart Foundation (Nederlandse Hartstichting) (Netherlands) (ref: CD 300006)
Primary sponsor	Academic Medical Centre (AMC) (Netherlands)
Secondary sponsor(s)	-
Contact for public queries	Paul J Nederkoorn; P.J.Nederkoorn@amc.uva.nl
Contact for scientific queries	Paul J Nederkoorn, Department of Neurology, Academic Medical Centre, PO box 22660, 1100 DD Amsterdam, The Netherlands.
Public title	Preventive Antibiotics in Stroke Study
Scientific title	Preventive ceftriaxone to improve functional health in patients with stroke by preventing infection: a multicentre prospective randomised controlled trial
Countries of recruitment	The Netherlands
Health condition(s) or problem(s) studied	Stroke, infection
Intervention(s)	Optimal medical care and ceftriaxone 2,000 mg intravenously, once daily, for four days, versus optimal medical care without ceftriaxone.
Key inclusion and exclusion criteria	Inclusion criteria: aged greater than or equal to 18 years, either sex; stroke (ischaemic and haemorrhagic); any measurable neurological deficit defined as National Institutes of Health Stroke Scale (NIHSS) greater than 1; stroke onset less than 24 hours; admission.
	Exclusion criteria: symptoms or signs of infection on admission requiring antibiotic therapy; use of antibiotics less than 24 hours before admission; pregnancy; hypersensitivity for cephalosporin; previous anaphylaxis for penicillin or derivates; subarachnoid haemorrhage; death seems imminent.
Study type	Multicentre prospective randomised open-label blinded end point trial
Date of first enrolment	4 July 2010
Target sample size	2,550
Recruitment status	Recruiting
Primary outcome(s)	Functional health at three-month follow-up, as assessed by the modified Rankin Scale (mRS)
Key secondary outcomes	Death rate at discharge and three months, infection rate during hospital admission; length of hospital admission; volume of post-stroke care; use of antibiotics during hospital stay; Quality adjusted life years (QALYs); costs.

**Table 2:** Protocol revision chronology

**Date**	**Protocol version and amendments**
5 May 2010	Original protocol
15 August 2010	Protocol version 1.1. Amendments: exclusion criterion ‘death seems imminent’ added; compulsory urine analysis and culture on admission omitted.
9 December 2010	Protocol version 1.2. Amendments: new study centres with new estimations of included patients were added; paragraph 6.6 ‘drug-accountability’: badge number of the administered ceftriaxone will be noted by the nurse administrating the medication into the ‘drug accountability form’ according to GCP-guidelines for pharmacies; paragraph 7.2 ‘randomisation, blinding and treatment allocation’: randomisation will not be stratified according to stroke type, solely by study centre and stroke severity; assessment of blinded outcome is specified as performed by a person not involved in the trial team; performance of interim analyses is specified as performed by an independent statistician not involved in the trial team; paragraph 8.2 ‘adverse and serious adverse events’: for each participating centre, a flowchart of serious adverse event/suspected unexpected serious adverse reactions (SAE/SUSAR) reporting will be provided in the local Investigator File; paragraph 8.5 ‘data monitoring’: reference to the monitoring plan is added.
10 January 2014	Protocol version 1.3. Amendment: change in primary analysis of primary outcome from dichotomised analysis to ordinal regression analysis according to the proportional odds model.
Total course of study	Participating centres were added (all participating centres are shown in Table 3).

**Table 3:** Centres participating in the Preventive Antibiotics in Stroke Study (PASS) with local investigators

**Centre**	**Local investigator**
Academic Medical Centre, Amsterdam	PJ Nederkoorn; D van de Beek
Albert Schweitzer Hospital, Dordrecht	H Kerkhoff
Amphia Hospital, Breda	MJM Remmers
Amstelland Hospital, Amstelveen	DSM Molenaar
Atrium Medical Centre, Heerlen	T Schreuder
Boven-IJ Hospital, Amsterdam	M Janmaat
Bronovo Hospital, The Hague	SM Manschot
Catharina Hospital, Eindhoven	K Keizer
Erasmus Medical Centre, Rotterdam	DWJ Dippel
Groene Hart Hospital, Gouda	K de Gans
HAGA Hospital, The Hague	SF de Bruijn
Kennemer Gasthuis, Haarlem	M Weisfelt
Laurentius Hospital, Roermond	ML van Goor
Martini Hospital, Groningen	ES Schut
Medical Centre Haaglanden, The Hague	K Jellema
Medical Centre Alkmaar	R ten Houten
Onze Lieve Vrouwe Gasthuis Amsterdam	JLM Bosboom
Orbis Medical Centre, Sittard	N van Orshoven
Rijnstate Hospital, Arnhem	SE Vermeer
Reinier de Graaf Hospital, Delft	LAM Aerden
Slotervaart Hospital, Amsterdam	ND Kruyt
Spaarne Hospital, Hoofddorp	ISJ Merkies
St. Franciscus Gasthuis, Rotterdam	FH Vermeij
University Medical Centre Radboud, Nijmegen	E van Dijk
University Medical Centre, Utrecht	HB van der Worp
VU Medical Centre, Amsterdam	MC Visser
Westfries Gasthuis Hoorn	TC van der Ree
Ijsselland Hospital, Capelle aan den IJssel	AD Wijnhoud
Zaans Medical Centre, Zaandam	RM van den Berg - Vos
ZGT, Almelo	LJA Reichman

The primary end point of the PASS is functional outcome at three-month follow-up on the modified Rankin Scale (mRS), a well-validated functional outcome scale in stroke patients [6]. In the protocol publication, the primary efficacy end point has been defined as the functional outcome at the three-month follow-up, as assessed by the mRS dichotomised as a favourable outcome (mRS 0 to 2) or as an unfavourable outcome (mRS 3 to 6). The proportional odds model was defined as the secondary analysis of the primary end point [5]. Secondary outcome measures were death rate at discharge and three-months, infection rate during hospital admission, length of hospital admission, volume of post-stroke care, use of antibiotics during follow-up, Quality-adjusted life years (QALYs); and costs. In this update publication of PASS, we change our primary outcome analysis from dichotomisation to ordinal regression analysis on the mRS. We also change the secondary outcome of use of antibiotics during follow-up into use of antibiotics during hospital stay.

### Change in primary analysis of primary outcome and adaptation of sample size

The modified Rankin Scale is a well-validated functional scale for assessing outcome after stroke. Analysis on a dichotomisation in favourable versus unfavourable outcome delivers easily comprehensible results. However, cut-off is arbitrarily and solely based on improvement beyond this one cut-off point. A secondary analysis including 55 datasets of stroke trials showed that statistical analysis based on the ordered nature of functional outcome data versus dichotomisation was more efficient and more likely to deliver reliable results [7]. Although there were some annotations regarding this publication, more and more studies are using ordinal regression analysis [8-10].

In the design of PASS, both dichotomisation and ordinal regression analysis were described as analysis of the primary outcome [5]. We based our initial sample size calculation on the dichotomised outcome (favourable versus unfavourable outcome). Dichotomisation was chosen as primary analysis of efficacy because of the widespread use in stroke trials [5,11]. However, trial completion will take an unrealistically long time with excessive costs with the current inclusion rate of 15 patients per week. Therefore, we now propose a switch in primary analysis of the primary outcome using an ordinal outcome analysis. The primary outcome will remain to be assessed on the mRS. The primary outcome with dichotomisation will be presented as secondary analysis of primary outcome. Using ordinal regression analysis for PASS enables us to preserve the assumptions of the strength of the treatment effect with a lower total sample size.

### Sample size

We based our initial sample size calculation on the dichotomised outcome (favourable versus unfavourable outcome). With the assumption of reduction of unfavourable outcome of 5%, with a power of 80% and *P*-value of 0.05, we aimed to include 3,200 patients.

**Figure 1:**
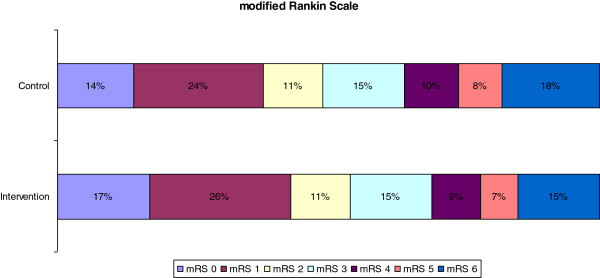
**Expected distribution of the two treatment arms.** The assumption for the distribution on mRS in the control-arm is based on the control-arm in the Paracetamol (Acetaminophen) In Stroke (PAIS) trial, which had almost similar inclusion criteria as PASS [9].

We now propose a new sample size of 2,550 patients, which is based on the ordinal regression analysis of the primary outcome. For this analysis we will use the ‘proportional odds model’, also known as the ‘cumulative logit model’ [12]. The assumption for the distribution on mRS in the control-arm is based on the control-arm in the Paracetamol (Acetaminophen) In Stroke (PAIS) trial, which had almost similar inclusion criteria as PASS [13]. We assumed a proportional odds ratio of 0.818 between all pairs of category groups, similar to the assumption in the original sample size calculation (odds ratio of 0.818 for mRS 0 to 2 versus mRS 3 to 6). Figure 1 shows the expected distribution of the two treatment arms. Using the method of Whitehead, with alpha 0.05 and power 80%, the desired sample size in the proportional odds model is estimated at a total of 2,410 patients [14]. Given an expected rate of patients lost to follow-up and/or patients with incomplete data of 5%, a conservative estimate for the new sample size with the primary end point analysed on all categories of the mRS is 2,531 patients. We will therefore adapt the sample size to 2,550 patients; a reduction of 650 patients compared to the original sample size estimate based on a dichotomous outcome on the mRS. This decision has been made by the researchers without any knowledge of outcome data per treatment group.

### Recruitment target

By 12 February 2014, 2,470 patients were included in the PASS. Up-to-date statistics can be found at http://www.passamc.nl. With a stable weekly inclusion rate of 15 patients, follow-up of the last included patient is expected in June 2014.

### Definitions of infection

Infection rate during hospital admission will be assessed in two ways. First, clinical diagnosis according to the treating physician will be recorded. Second, diagnosis of infection will be judged by two experienced infectious diseases specialists, blinded for treatment allocation, using the modified criteria of the United States Centres for Disease Control and Prevention [15]. This will be done in all patients who developed fever or a new onset delirium during admission, in patients in whom there was suspicion of infection but no diagnostics were performed, and in patients in a palliative care setting. One important issue that needs to be addressed is the risk of performance and detection bias. Since the treating physician is aware of the treatment allocation, this could influence decisions on non-scheduled treatment. For the PASS, the most important issue to address is the detection and treatment of infection. A physician could be more or less likely to order investigations or start treatment for a possible infection depending on the treatment allocation. By giving recommendations for diagnostic procedures in the previously mentioned subgroups of patients, and by collecting results of these procedures in standardized case record forms, we try to limit this form of bias.

### Monitoring of antibiotic resistance

One of the most important mechanisms of resistance against third generation cephalosporins is forming of extended-spectrum-β-lactamase (ESBL), an enzyme that renders antibiotics ineffective, in *Enterobacteriaceae*. In our study we monitor the prevalence of ESBL-producing bacteria in both treatment arms. We therefore collect stool specimens at admission and discharge in a subgroup of patients. To date, samples have been obtained in 300 patients.

### Development of the statistical analysis plan

Currently, the statistical analysis plan is being finalised, without insight in to the unblinded data. It will be published before the randomisation code is broken in late 2014. The statistical analysis plan describes the analysis of primary outcome with ordinal regression analysis and a secondary dichotomised analysis into detail. It also describes a small number of prespecified subgroup analyses, and a larger number of exploratory secondary analyses, that will be performed, as well as treatment of missing values.

## Discussion

The PASS aims to investigate whether preventive antibiotic therapy improves functional outcome by preventing infections. The results of a trial examining the effect of preventive antibiotic therapy on functional outcome are urgently warranted. Infection after stroke is common and infection has repeatedly been shown to worsen outcome [1,2,16,17]. Since previous studies on preventive antibiotic therapy were too small, heterogeneous, or did not investigate functional outcome, no sufficient information is available on the role of preventive antibiotic therapy in acute stroke [4].

With this update of the protocol, we present a change in primary analysis of the primary outcome on the mRS from a dichotomised analysis to an ordinal regression analysis. Ordinal analysis of outcome data is increasingly common in acute stroke trials as well as in other trials, for example on traumatic brain injury [7,18]. Data is used more efficiently with ordinal analysis as compared to a dichotomised analysis. For example the ECASS-II trial failed to show an effect of treatment in the dichotomised approach, but did show an affect with ordinal shift analysis [19].

Different approaches can be used for the analysis of ordinal outcome data. In the PASS we chose the ordinal regression analysis as primary analysis of outcome, which was already described as a secondary analysis of primary outcome in the original protocol. The proportional odds model provides additional information from ordinal outcome data, as it takes into account improvements at any point on the mRS [18]. This method is highly efficient when compared to a dichotomised approach, but also when compared to other ordinal approaches [18]. A possible disadvantage of this approach is the assumption of proportional odds across all groups. In PASS, we chose this method because we expected a similar effect of preventive antibiotic treatment across all outcomes, and therefore expect to meet the assumptions of the proportional odds model.

With the new sample size of 2,550 patients we expect completion of inclusion of patients in PASS in June 2014.

## Abbreviations

AMC: Academic Medical Centre; ESBL: extended-spectrum-β-lactamase, mRS, modified Rankin Scale; NIHSS: National Institutes of Health Stroke Scale; PASS: Preventive Antibiotics in Stroke Study; PROBE: Prospective, Randomised, Open-label, Blinded End point; SAE: serious adverse event; SUSAR: suspected unexpected serious adverse reaction.

## Competing interests

All authors declare not to have any competing interests.

## Authors’ contributions

WFW, JDV performed conception and design of the work and drafted the work. PJN and DvdB performed conception and design of the work and revised it critically for important intellectual content. NG performed the statistical analysis for this paper and revised it critically. DWJD, MGWD, TvdP, JMP, LS, FHV were involved in design of the work, and revised it critically for intellectual content. All authors approved the final version.
